# Work of Medical Missions

**Published:** 1923-06

**Authors:** 


### WORK OF MEDICAL MISSIONS.

THE heroic rescue by Mrs. Starr of Miss Ellis from the tribesmen on the Afghan border has reminded the English public that there are to-day in various parts of the world such people as medical missionaries. What Mrs. Starr, of the Church Missionary Society, had done, said the Bishop of Dover at the recent annual meeting of the Medical Missions Department of the Society for the Propagation of the Gospel, held at the Church House, Westminster, had thrilled the whole of England, and would make everybody feel an intelligent interest in the work of medical missions. Certainly the S. P. G. medical mission branch has a remarkable record of achievement for last year. There are hospitals at Delhi, Kamdara, Irungalur, Ramnad and Mandalay. At Singapore the foundation stone of a new hospital has been laid, and in Northern Shansi the building of another hospital is proceeding. At Durban an operating theatre has been added to St. Aidan's Hospital, and in St. John's, Kaffraria, the hospital buildings are practically completed; it is now possible to take in patients and perform operations. At all these centres, however, activity is hampered by the smallness of medical staffs.

### The Need of Sufferers Abroad.

"You do not meet anyone," said the Bishop of Dover, "who makes any objection to medical missions. They make their appeal to everybody." If the need of hospital work at home made an appeal that few could resist, how much greater was the appeal made for the needs of people abroad, where they had none of the knowledge even of those elements of nursing and of medicine which nearly everybody possessed in a country like England? It hurt one at home to think of the accumulated unnecessary suffering that was being borne by men and women all over the world. "We indeed feel that we are called upon to do all in our power to relieve that mass of suffering by providing medical missions." Some practical counsel as to home organisation work was given by Miss A. Potts, Honorary Secretary for Medical Missions in Newcastle Diocese since 1914. Mr. B. L. E. Sigamoney, an Indian mission worker of St. Aidan's, Durban, followed with an interesting account of life and work in that part of South Africa. An address by the Rev. T. R. G. Lyell, Deputation Secretary of the Medical Missions Department, and late district magistrate at Bagdad completed the programme.

### More Doctors Wanted.

In 1922 the income of the Medical Missions Department of the Society was £21,540. New requests are continually being made from different parts of the world for doctors; some of them are of peculiar urgency, several being needed to prevent breakdown of workers. Women doctors are offering themselves, and it is stated that although some of the needs can only be met by men, women can fill many posts. Offers of service, however, have almost passed the number of those who can be supported by the available income.

